# Global and regional estimates of the morbidity due to type I diabetes among children aged 0-4 years: a systematic review and analysis

**DOI:** 10.7189/jogh.08.021101

**Published:** 2018-12

**Authors:** Davies Adeloye, Kit Yee Chan, Natasha Thorley, Charlotte Jones, David Johnstone, Ari L'Heveder, Vanja Saftic, David Henderson, Mickey Chopra, Harry Campbell, Igor Rudan

**Affiliations:** 1 Centre for Global Health Research and World Health Organization’s Collaborating Centre for Population Health, Research and Training, The Usher Institute, University of Edinburgh, UK; 2 Child and Youth Protection Center of Zagreb, Croatia; 3 Croatian Catholic University, Zagreb, Croatia; 4 The World Bank Group, Washington, D.C., USA

## Abstract

**Background:**

Epidemiology of type 1 diabetes mellitus (T1DM) among children aged 0-4 years globally is not well understood. We aim to assess the incidence of T1DM in low- and middle-income countries (LMIC) by conducting a systematic review of previous reports. We also aim to address possible contribution to child mortality and to identify any temporal trends.

**Methods:**

A systematic review was performed using a carefully designed search strategy to explore MEDLINE, EMBASE and Global Health databases. Data was extracted from all studies that satisfied the inclusion criteria –a total of 83 records extracted from 26 830 sources that were analysed. We used the Grading of Recommendations Assessment, Development and Evaluation (GRADE) process to assess quality of evidence and applied meta-analysis approaches to assess global and regional incidence and time trends.

**Results:**

The overall pooled incidence of T1DM in children aged 0-4 years globally is 11.2 (95% CI = 10.0-12.3) per 100 000 child years. The regional incidence were the highest for European Region A (EUR A) at 15.5 (95% CI = 13.5-17.5) per 100 000 child years. EUR C had the incidence of 10.0 (95% CI = 6.5-13.6) and EUR B 5.8 (95% CI = 4.7-7.0), Region of the Americas A (AMR A) 11.4 (95% CI = 7.8-14.9), AMR B of 2.5 (95% CI = 0.2-4.8), Eastern Mediterranean Region (EMR B) 7.1 (95% CI = 4.2-10.0) and Western Pacific Region (WPR A) 7.0 (95% CI = 2.9-11.0) per 100 000 child years, while other regions had very low rates or no data. When data points were categorised in the study periods and re-analysed, an increasing trend of the T1DM incidence was observed, with the incidence of 20.9 (95% CI = 7.8-34.1) per 100 000 child years in the years 2010-2015, preceded by 13.2 (95% CI = 11.0-15.5) in 2000-2009 study period, 10.0 (95% CI = 8.4-11.7) in 1990-1999 and 8.3 (95% CI = 5.1-11.6) in 1980-1989, respectively. Although the data are scarce, and variation and uncertainty are large, we estimated that the number of new cases of T1DM among children aged 0-4 years in the world each year is between 100 000 and 150 000.

**Conclusions:**

The identified large variation in incidence estimates for different parts of the world, along with scarcity of information and the identified strong temporal increase in T1DM incidence suggest a clear need for further research into this subject.

The epidemiology of type 1 diabetes mellitus (T1DM) is increasingly studied across several world regions to provide better understanding of the disease in these settings and prompt relevant public health and policy response [1,2]. According to the International Diabetes Federation (IDF) atlas, about 151 million people were estimated to have any type of diabetes globally in 2000 [3], 194 million in 2003 [4], 246 million in 2006 [5], 415 million in 2015 and a projected increase to 642 million by 2040 [6,7]. Mortality from diabetes globally increased from 16.3/100 000 to 19.5/100 000, and disability adjusted life years (DALYs) also increased from 523/100 000 to 680/100 000 between 1990 and 2015 respectively [8,9].

T1DM remains a large public health concern globally, especially due to fast rising and varying incidence reported across diverse population groups [10]. According to the WHO Multinational Project for Childhood Diabetes (DiaMond) Research Group, overall age-adjusted incidence of T1DM varied from 0.1 in China and Venezuela to 37 per 100 000 person-years in Sardinia and in Finland, accounting for over 350-fold difference in the T1DM incidence in about a hundred different populations of children aged 0-14 years studied worldwide in early 1990s [11]. Experts have reported that this marked variation may reflect the heterogeneity within and between major ethnic populations globally [12,13], which possibly account for the hereditary and genetic factors indicated in the aetiology of T1DM. For example, T1DM incidence was reported to be higher among populations of white European ancestry, as observed in Nordic populations of Finland, Sweden, and Norway compared to other world regions [11]. In Israel, marked ethnic variations were observed, especially between Jewish and Arab populations [11]. In Africa, geographical and ethnic variations were observed in some populations in Tanzania, Nigeria, South Africa, and Ethiopia [14-16]. Although, genetic factors have been indicated in the development of T1DM, it is important to note that the aetiology is actually multifactorial, for which several theories have been suggested [17].

Meanwhile, increasing incidence and mortality from T1DM has been reported in children and adolescents, especially in Central America, West Indies and several European countries [13]. A statistically significant increase in T1DM incidence of 65% was noted in all the population that were studied, mostly in the younger age groups [18]. In Europe, annual increment in incidence of T1DM in children ranged from 0.6%-15% across the 36 EURODIAB centres in the period 1989-2003 [10]. Moreover, in the US multicentre Search for Diabetes in Youth Study (SEARCH), there was marked annual increase in the incidence of T1DM in non-Hispanic White younger than 14 years, with a mean of 27.5 per 100 000 per year [19].

Patterson and colleagues provided worldwide estimates of T1DM in children aged 0-14 years, and highlighted challenges in the collation of epidemiological reports on T1DM in this age group [12]. There are currently few reports on deaths from T1DM, especially from Africa and many low- and middle-income countries (LMICs) [20]. Major causes of deaths have been linked to poor diagnosis and treatment, with many presenting with acute complications, infections, cardiovascular diseases and chronic kidney diseases [2], with many calling for improved and continuous strategies for disease prevention, diagnosis and treatment on a population-wide scale [21]. In fact, speculations were reported hypothesising that T1DM could perhaps explain a major part of the unclassified causes of 0-14 years mortality in many low-resource settings [22]. In the light of the relatively poor understanding of the burden of T1DM in LMICs, especially among children under five years, this study aimed to review available evidence on T1DM in children aged 0-4 years globally in order to contribute to the understanding of the epidemiology, and provide improved estimate of the incidence of the disease in this age group.

## METHODS

### Search strategy

After identification of relevant Medical Subject Headings (MESH) and keywords, a final search strategy was developed. Searches were conducted on three main databases: Medline, EMBASE and Global Health. Literature searches were conducted on 15 August 2018.The search date was set from January 1980 to date (Tables S1-S3 in **Online Supplementary Document(Online Supplementary Document)**).

### Selection criteria

We included i) studies published from 1980 to 2017 globally that referred to diabetes among children, ii) studies that directly attempted to estimate the prevalence, incidence and/or under-five mortality from T1DM across various world regions, and iii) studies that provided information that helps to understand the determinants of the occurrence and aetiology, clinical features, management, case-fatality rates, and outcomes of T1DM in children.

We excluded i) studies that are reviews, viewpoints and commentaries, iii) studies that do not report relevant denominators from which prevalence, incidence and/or under-five mortality from T1DM can be estimated, iv) studies with ambiguous study designs and analysis, v) studies without active follow-up periods, and vi) studies with case definitions not clearly defined and consistently applied.

### Case definitions

In this review, we included studies that clearly reported T1DM diagnosis (based on registries and a clinical diagnosis of type 1 diabetes). Case definitions employed across studies varied, including the WHO criteria [23,24], the American Diabetic Association (ADA) criteria [25,26], and the WHO-IDF recommendation diagnosis of diabetes [27]. The final case definitions employed in the selection criteria were as follows:

- Fasting plasma glucose level at or above 7.0 mmol/L (126 mg/dL);
  - Symptoms of hyperglycemia and casual plasma glucose at or above 11.1 mmol/L (200 mg/dL).
    - Glycated hemoglobin (hemoglobin A1C) at or above 48 mmol/mol (≥ 6.5 Diabetes Control and Complications Trial DCCT % [28] (**Table 1**).

**Table 1:** Diagnostic criteria for diabetes*

Condition (Unit)	Casual glucose mmol/l(mg/dl)	Fasting glucose mmol/l(mg/dl)	HbA_1c_ (mmol/mol)	DCCT %
Normal	<7.8 (<140)	<6.1 (<110)	<42	<6.0
Impaired fasting glycaemia	<7.8 (<140)	≥6.1(≥110) &<7.0(<126)	42-46	6.0–6.4
Impaired glucose tolerance	≥7.8 (≥140)	<7.0 (<126)	42-46	6.0–6.4
Diabetes mellitus	≥11.1 (≥200)	≥7.0 (≥126)	≥48	≥6.5

mmol/l(mg/dl) – millimoles per litre (milligrams per decilitre), HbA1**_c_** – Hemoglobin A1c, DCCT – Diabetes Control and Complications Trial.

*Source: [24-28].

### Quality criteria and grading

Quality of individual studies was assessed based on the following criteria:

- Study design: Under this, flaws in the design and execution of study were examined. Basically, this assesses methods of estimation of sample size and sampling methods across studies (if necessary), and the methods of dealing with design specific issues such as: training of study investigators, adherence to standardized protocol and case definitions/ascertainment for determining T1DM
- Study analysis: This assesses the appropriateness of statistical and analytical methods employed across studies;
  iii Study limitations: This assesses if the study explicitly stated the limitations, as this may further guide in the choice of selection; and
    - Generalizability to a larger population: This broadly assesses if the sample size or study population was representative of a larger population in the region of study.

For the quality grading, the Grading of Recommendations Assessment, Development and Evaluation (GRADE) guidelines [29] were adapted:

- High-quality: Studies with all four criteria, or any three including “study design” highlighted above, well represented;
- Moderate-quality: Studies with any three of the four criteria, or any two including “study design” highlighted above, well represented;
- Low-quality: Studies with any two of the four criteria or “study design” only, highlighted above, well represented; and
- Very low-quality: Studies with only one (excluding “study design”) or none of the four criteria highlighted above, well represented.

As a basic rule, all studies that were graded as high and moderate quality were included in the quantitative analysis. Some low-quality studies were also included in the quantitative analysis on the basis of good study designs, and details of these studies will be described further in the discussion section. However, all very low-quality studies have been excluded.

### Data extraction

To allow for consistency, parallel searches were conducted following a predesigned guideline. Disagreements in study selections were resolved by consensus. Data were abstracted systematically on period of study, location, study population, mean age or age range, number of T1DM cases, incidence, prevalence (if reported), and number of deaths from T1DM (if reported). All data were categorised by the World Health Organization (WHO) regions and study periods for further analysis. For studies conducted on the same study site, population or cohort, the first chronologically published study was selected, and all additional data from other studies were included in the selected paper.

### Data analysis

A random-effects meta-analysis (DerSimonian and Laird method) was conducted on extracted crude incidence rates [30]. This was reported separately for the WHO regions and study periods, all expressed per 100 000 child years (population data were as reported in studies). Standard errors were determined from the reported crude estimates and child-years of follow-up, assuming a binominal (or Poisson) distribution. Heterogeneity between studies was assessed using I-squared (I^2^) statistics, and subgroup analysis was conducted to detect causes of heterogeneity. Weights were based on reported study population size, and data from multiple study sites were combined. However, estimates from countries with data available were only extrapolated to for the WHO region of those countries, and not individually for other countries in the same region. There were no data points on deaths in under-fives, most T1DM paediatric studies that reported deaths were in the age 0-14 years and did not provide breakdowns to allow extraction of deaths in under-fives. Based on published statistical evidence on chronic diseases, we assumed the prevalence of T1DM at any point in time to be about five times greater than the incidence [31]. In addition, based on the assumption of stable demographic changes in children aged 0-4 years globally, number of T1DM incident cases was extrapolated from the meta-estimates for the years 1990, 2010, and 2015 using the United Nations (UN) population projections [32]. All statistical analyses were conducted on STATA (Stata Corp V.15, College Station, Texas TX, USA). This study was conducted in line with the Preferred reporting items for systematic reviews and meta-analyses (PRISMA) statement.

## RESULTS

### Literature search

**Figure 1:**
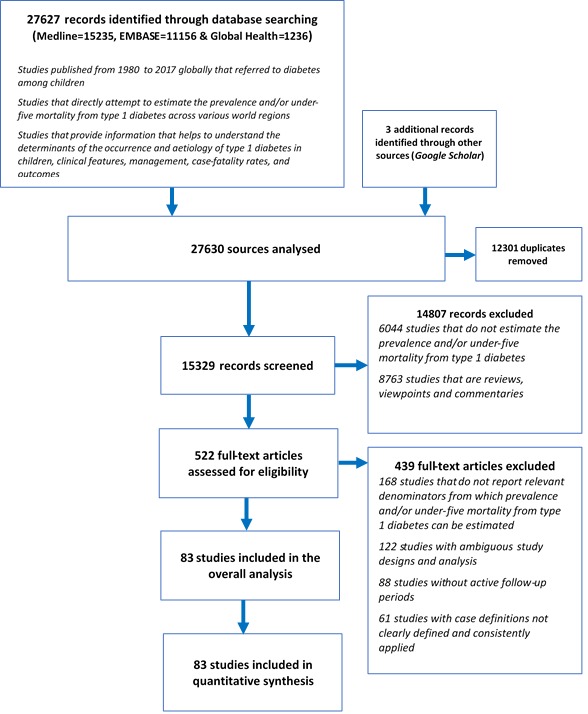
Flowchart of study selection.

A total of 27 627 records were identified through database searching (Medline 15 235, EMBASE 11 156 and Global Health 1236) and 3 additional records were obtained from Google Scholar, resulting in a total of 27 630 records analysed. After removing duplicates, 15 329 records remained. Of these, 14 807 records were excluded, giving a total of 522 full-text articles assessed for eligibility. Then, 439 full-text articles were further excluded. A total of 83 studies were finally included for the analysis [10,11,13,17,19-23,33-106] (**Figure 1****,** and Table S4 in **Online Supplementary Document(Online Supplementary Document)**).

### Study characteristics

The numbers of studies conducted in declining order by decade were: 1990-1999 (36 studies), 2000-2009 (28 studies), 1980-1989 (14 studies), and 2010-2017 (5 studies). Most of the studies – 56 in total – were conducted in the European region (EUR A-39, EUR B-14, EUR C-3). Eastern Mediterranean Region (EMR) contributed 11 studies, American region (AMR) 8 studies, Western Pacific region 6 studies, while South East Asian (SEAR) and African regions (AFR) had only 1 study each. About two-thirds of selected studies were rated as high-quality with most of these (70.5%) conducted in the European region. See Appendix S1 in **Online Supplementary Document(Online Supplementary Document)** for the full list of studies and study characteristics.

### Incidence rates of T1DM

**Figure 2:**
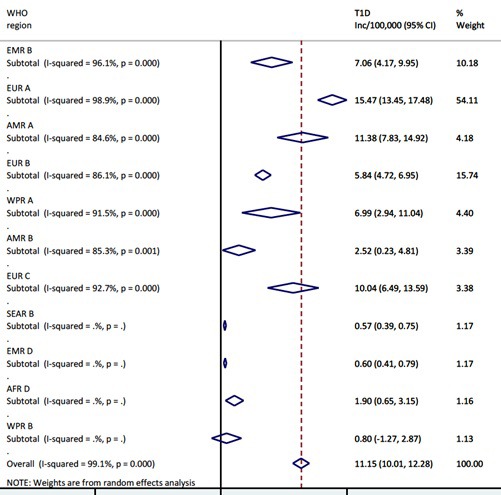
Meta-estimate of the incidence rate of type 1 diabetes mellitus by WHO regions.

**Figure 3:**
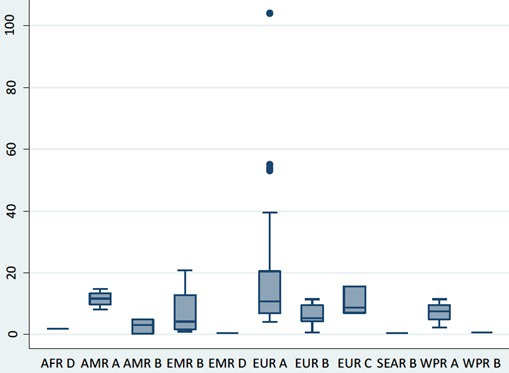
Box and whisker plot of the incidence rate of type 1 diabetes mellitus by WHO regions.

The overall pooled incidence of T1DM in children aged 0-4 years globally is 11.2 (95% CI = 10.0-12.3) per 100 000 child years. Heterogeneity was high across studies, with I^2^ estimated at 99.1%, *P* < 0.000. The regional estimates showed that European region had the highest pooled incidence, with EUR A leading all regions at 15.5 (95% CI = 13.5-17.5) per 100 000 child years. However, EUR C had 10.0 (95% CI = 6.5-13.6) per 100 000 child years, and EUR B 5.8 (95% CI = 4.7-7.0) per 100 000 child years, ie, considerably lower rates. The American region also showed variation, with AMR A incidence of 11.4 (95% CI = 7.8-14.9) and AMR B of 2.5 (95% CI = 0.2-4.8) per 100 000 child years, respectively. High incidence rates were recorded in EMR B with 7.1 (95% CI = 4.2-10.0) and WPR A with 7.0 (95% CI = 2.9-11.0) per 100 000 child years, respectively (**Figure 2** and **Figure 3**).

**Figure 4:**
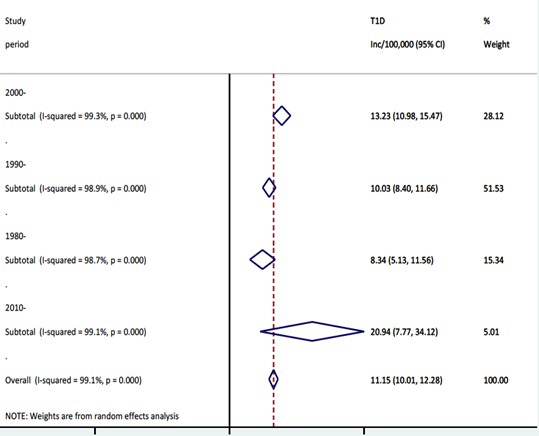
Meta-estimate of the incidence rate of type 1 diabetes mellitus by study period.

**Figure 5:**
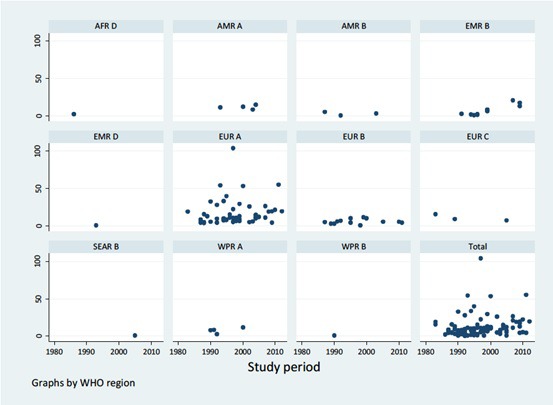
Incidence rate of type 1 diabetes mellitus by study period.

When data points were categorised according to the study periods and re-analysed, an increasing trend of the T1DM incidence was observed (**Figure 4** and **Figure 5**). The highest pooled incidence of T1DM was recorded in the years 2010-2015, with 20.9 (95% CI = 7.8-34.1) per 100 000 child years. This was preceded by the 2000-2009 study period, with an incidence rate of 13.2 (95% CI = 11.0-15.5) per 100 000 child years. The years 1990-1999 and 1980-1989 showed pooled incidence of 10.0 (95% CI = 8.4-11.7) and 8.3 (95% CI = 5.1-11.6) per 100 000 child years, respectively. Using the United Nations population projection, and assuming demographic factors and differences between world regions were fully accounted for, these rates would amount to about 53 000 new cases in children aged 0-4 years in 1990, 62 000 new cases in 2000, 84 000 new cases in 2010, and 136 000 new T1DM cases in 2015, respectively. Given that very few deaths are identified in this age group (although the data on mortality in 0-4 year group are remarkably scarce), the prevalence at any point in time should be expected to be about 5 times greater than the incidence, ie, to range from about 300 000 cases in 1990 to just under 700 000 in 2015. Still, there is a very considerable uncertainty about these estimates, with hardly any understanding of case-fatality rate in this period.

In order to speculate about the mortality from T1DM in the age group 0-4 years, we could apply historic mortality rates from the 19th century and early 20th century from USA and Scandinavian countries, which were in range of 1-4 per 100 000 per child years (these rates were relevant to the age group of 0-14 years).

If those rates were applied to the global child population aged 0-4 years today, they would result in 6500 to 26 000 child deaths. However, given the near-certain fact that those deaths were predominantly occurring after year 4, and that there are treatment options currently available, it would be surprising if the number of deaths in this period globally amounted to a four-digit number, rather it would likely be measured in hundreds.

## DISCUSSION

This study, to the best of our knowledge, provides the first systematically derived global and regional estimate of the incidence of type 1 diabetes among children aged 0-4 years. Prior to the conduct of this study, there have been some efforts to describe the burden of T1DM in children globally, notably the WHO DiaMond project, which used standardized type 1 diabetes incidence registries worldwide [11]. Despite using a capture-recapture method to ascertain completeness of registry, one major limitation of this study was relative under-reporting and under-estimation of most registries, especially in developing countries, where secondary sources of case ascertainment are largely unavailable. Indeed, few studies on the epidemiology of T1DM exists globally, most of these focus on clinical diagnosis and treatment, and are generally on adolescent populations [107]. In view of current reports suggesting an increased prevalence or incidence in younger population groups [108], an understanding of the number of cases in children, especially under-fives, which this current study is providing, may be worthwhile.

In this study, the overall pooled incidence of T1DM in children aged 0-4 years globally in 2015 was 20.9 (7.8-34.1) per 100 000 child years, accounting for about 136 000 T1DM cases. Our findings are higher than previously reported, and may further support recent reports that increasing incidence of T1DM is shifting towards younger age groups [108]. In the DiaMond study, a total of 19 164 T1DM cases in children aged 0-14 years were identified in over 100 centres with age-adjusted incidence ranging from 0.1-36.8 per 100 000/y [11].

A consistently increasing incident cases of T1DM was further observed over the study period, with about 53 000, 62 000, 84 000, and 136 000 T1DM cases estimated in children aged 0-4 years in 1990, 2000, 2010 and 2015, respectively. This finding, although significantly higher than previous estimates, is supported by many studies. In the WHO DiaMond study, between 1990 and 1994, the average increase in the incidence of T1DM was 2.4%, with this increasing to 3.4% the during the years 1995-1999. Patterson and colleagues predicted the global prevalence of T1DM will increase from 94 000 in 2005 to 160 000 in 2020, with this likely doubling in children younger than five years [10]. Onkamo et al. also reported that the incidence of T1DM is increasing in low and high incidence populations, and that by the year 2010, the incidence in many other populations may be over 30 per 100 000/y [18].

Meanwhile, we observed our 2010 and 2015 estimates are higher than annual type 1 diabetes cases of 77 880 and 96 100 in children 0-14 years reported by IDF in 2011 and 2017, respectively [109]. We believe, this contrast, is due to our subgroup-analysis by study period, which clearly showed an increasing trend. Several authors have suggested that the global increase over the years in the incidence of T1DM may be due to better case ascertainment and registration process across many developing countries [2,13]. However, without a subgroup-analysis, our overall pooled estimate from all studies at 11.2 per 100 000 would account for about 73 000 cases of T1DM in children under 5 years in 2015, which may not be too far from the IDF estimates.

From the regional estimates, the European region had the highest T1DM incidence rate with EUR A leading all regions at 15.5 (13.5-17.5) per 100 000 child years, and this is followed by the American region, with AMR A having 11.4 (7.8-14.9). According to the WHO DiaMond study, higher incidence rates of T1DM have been observed Europe and North America, mostly due to the fact that most registries in these regions have been established as far back as the 1980s, and are therefore better equipped to collate relatively complete data on T1DM than other world regions [110]. As noted in the introduction, geographical and climatic factors may have also influenced distribution of mean incidence rates of T1DM globally [111,112]. Emerging evidence also suggests that environmental factors are now able to initiate the development of T1DM in genotypes previously not known to be associated with the disease [2]. However, some experts have stated that geographical and environmental influences do not overtly account for the rapidly increasing incidence of T1DM [113,114].

As observed in this study, some studies have also noted that data from African and South East Asia are still relatively sparse, thus making the understanding of T1DM in the regions quite difficult [11,107]. These are therefore regions requiring further epidemiological surveys and research to contribute to better understanding of T1DM globally.

This study could have been limited by a number of factors. However, one major constraint, which has already been reported in previous studies, is that most of the publications in this review were from the European region. This implies that estimates may reflect more the conditions in these settings, and may not be generalizable to other world regions. Moreover, with Africa having only one study, it may be important to raise more awareness in this region on T1DM among children aged 0-4 years, especially to support efforts (including availability of insulin) targeted at regions with very low national income, large child populations and poor child health outcomes. Additionally, there were no studies that reported mortality and case fatality rates from T1DM in children aged 0-4 years, as this would have contributed significantly to the understanding of its epidemiology and instituting necessary intervention measures. Largely, type 1 diabetes seems to be another major gap in global child health estimates. While the prevalence and incidence patterns across many child populations appear to be unfolding, it still remains unclear if this would contribute to increase child mortality, especially in the 0-4 years age group. It is however certainly worth addressing in terms of morbidity and mortality in LMICs, as new information is beginning to emerge.
