# Supplementary material for: Global and regional estimates of the morbidity due to type I diabetes among children aged 0-4 years: a systematic review and analysis
Source: J Glob Health. 2018 Sep 26;8(2):021101. doi: 10.7189/jogh.08.021101 (PMC6214490; doi:10.7189/jogh.08.021101)
Supplement: Online Supplementary Document [file jogh-08-021101-s001.pdf]

Online Supplementary Document

Adeloye et al. Global and regional estimates of the morbidity due to type I diabetes among children aged 0-4 years: a systematic review and analysis

J Glob Health 2018;8:021101

Table S1. Type 1 Diabetes Search Terms (MEDLINE)

#	Searches	Hits
1	exp glucose metabolism disorders/ or exp diabetes mellitus/ or exp diabetes mellitus, type 1/ or exp diabetes mellitus, type 2/ or exp diabetes, gestational/ or exp diabetic ketoacidosis/ or exp prediabetic state/ or exp glycosuria/ or exp hyperglycemia/ or exp glucose intolerance/	394634
2	exp vital statistics/ or exp incidence/	689447
3	(incidence* or prevalence* or morbidity or mortality).tw.	1296484
4	(disease adj3 burden).tw.	11981
5	exp "cost of illness"/	18825
6	exp quality-adjusted life years/	7483
7	QALY.tw.	4182
8	Disability adjusted life years.mp.	983
9	(initial adj2 burden).tw.	194
10	exp risk factors/	593406
11	2 or 3 or 4 or 5 or 6 or 7 or 8 or 9 or 10	1990285
12	1 and 11	90868
13	limit 12 to (yr="1990 -Current" and "all child (0 to 18 years)")	15235

Table S2. Type 1 Diabetes Search Terms (EMBASE)

#	Searches	Hits
1	exp glucose metabolism disorders/ or exp diabetes mellitus/ or exp diabetes mellitus, type 1/ or exp diabetes mellitus, type 2/ or exp diabetes, gestational/ or exp diabetic ketoacidosis/ or exp prediabetic state/ or exp glycosuria/ or exp hyperglycemia/ or exp glucose intolerance/	784984
2	exp vital statistics/ or exp incidence/	282502
3	(incidence* or prevalence* or morbidity or mortality).tw.	1831362
4	(disease adj3 burden).tw.	19994
5	exp "cost of illness"/	15304
6	exp quality-adjusted life years/	13770
7	QALY.tw.	8215
8	Disability adjusted life years.mp.	1466
9	(initial adj2 burden).tw.	291
10	exp risk factors/	673443
11	2 or 3 or 4 or 5 or 6 or 7 or 8 or 9 or 10	2393629
12	1 and 11	208326
13	**limit 12 to (yr="1990 -Current" and child <unspecified age>)**	11156

Table S3. Type 1 Diabetes Search Terms (GLOBAL HEALTH)

#	Searches	Hits
1	exp glucose metabolism disorders/ or exp diabetes mellitus/ or exp diabetes mellitus, type 1/ or exp diabetes mellitus, type 2/ or exp diabetes, gestational/ or exp diabetic ketoacidosis/ or exp prediabetic state/ or exp glycosuria/ or exp hyperglycemia/ or exp glucose intolerance/	36713
2	exp vital statistics/ or exp incidence/	121400
3	(incidence* or prevalence* or morbidity or mortality).tw.	391951
4	(disease adj3 burden).tw.	6599
5	exp "cost of illness"/	0
6	exp quality-adjusted life years/	0
7	QALY.tw.	799
8	Disability adjusted life years.mp.	830
9	(initial adj2 burden).tw.	24
10	exp risk factors/	132753
11	2 or 3 or 4 or 5 or 6 or 7 or 8 or 9 or 10	470049
12	1 and 11	16652
13	children/	185106
14	12 and 13	1236
15	limit 14 to yr="1990 -Current"	1236

Table S4. Selected studies from all searches

Author	Location	WHO region	Period of study	Incident cases (0-4 years)	Child years at risk (0-4 years)	Incidence per 100000 child years
**Abduljabbar et al**	Dharan, Saudi Arabia	EMR B	Jan 1990 - Dec 2007	91	439189	20.72
**Abdul-Rasoul et al**	Kuwait, Farwania	EMR B	Jan 1995 - Dec 1999	10	24610	8.1
**Ajlouni et al**	Jordan, Nationwide	EMR B	Jan 1992 - Dec 1996	39	3000000	1.3
**Altobelli et al**	Italy, Abruzzo region	EUR A	Jan 1990 - Dec 1995	28	366552	7.63
**Arpi et al**	Catania, Sicily	EUR A	Jan 1989 - Dec 1998	65	707291	9.19
**Bahíllo Curieses et al**	Castila Y Leon, France	EUR A	Jan 2003 - Dec 2004	26	88272	14.7
**Barat et al**	France, Aquitaine	EUR A	Jan 1988 - Dec 1997	84	80764	104.01
**Bizzarri et al**	Rome-Lazio, Italy	EUR A	Jan 2004 - Dec 2009	175	4243618	4.12384
**Blanchard et al**	Manitoba, Canada	AMR A	Jan 1985 - Dec 1993	66	594595	11.1
**Bratina et al**	Slovenia, Nationwide	EUR A	Jan 1990 - Dec 1998	59	106181	6.17
	Tuzla, Bosnia and Herzegovenia	EUR B	Jan 1990 - Dec 1998	3	41618	0.8
**Bruno et al**	Turin, Italy	EUR A	Jan 1990- Dec 1994	43	438776	9.8
		EUR A	Jan 1995 - Dec 1999	28	444444	6.3
		EUR A	Jan 2000 - Dec 2004	47	465347	10.1
	Piedemont, Italy	EUR A	Jan 1990- Dec 1994	70	833333	8.4
		EUR A	Jan 1995 - Dec 1999	56	835821	6.7
		EUR A	Jan 2000 - Dec 2004	102	879310	11.6
**Campbell-Stokes**	New Zealand, Nationwide	WPR	Jan 1999 - Dec 2000	64	279603	11.44

**et al**		A				
**Carle et al**	Italy (North, Central, South, Sardegna)	EUR A	Jan 1990 - Dec 1999	855	10055814	8.50
**Carrasco et al**	Chile, Santiago	AMR B	Jan 1986 - Dec 2003	257	8132911	3.16
**Casu et al**	Italy, Sardinia	EUR A	Jan 1989 - Dec 1999	256	876712	29.2
**Cepedano Dans et al**	Spain, Galicia	EUR A	Jan 2001 - Dec 2002	29	619446	4.68
**Cherubini et al**	Italy, Marche	EUR A	Jan 1990 - Dec 1992	8	60391	4.4
**Cinek et al**	Czech Republic	EUR A	Jan 1990 - Dec 1997	281	4762712	5.9
**Dahlquist et al**	Sweden, Nationwide	EUR A	Jan 1988 - Dec 1992	445	2816456	15.8
		EUR A	Jan 1993 - Dec 1997	648	2867257	22.6
		EUR A	Jan 1998 - Dec 2002	604	2336557	25.85
		EUR A	Jan 2003 - Dec 2007	663	2497175	26.55
**Demirbilek et al**	Turkey, Diyarbakir	EUR B	June 2010 - 31 May 2011	8	187592	4.3
**Dominguez**	Spain, Canary Island	EUR A	Jan 1995 - Dec 1996	27	176471	15.3
**Dziatkowiak et al**	Poland, Cracow, Wroclaw & Warsaw	EUR B	Jan 1987 - Dec 1999	132	2826552	4.67
**Feltbower et al**	UK, Yorkshire	EUR A	Jan - Dec 1990	124	2450593	5.06
		EUR A	Jan - Dec 1995	163	1899767	8.58
**Ferreira et al**	Brazil, Sao Paulo	AMR B	Jan 1987 - Dec 1991	8	163265	4.9
**Formosa et al**	Malta, Nationwide	EUR A	Jan 2006 - Dec 2010	22	101382	21.7
**Frongia et al**	Italy, Sardinia	EUR A	Jan 1993 - Dec 1996	15	27898	53.8
**Goday et al**	Spain, Catalonia	EUR A	Jan 1987 - Dec 1990	59	1340909	4.4
**Galler et al**	Germany, Saxony	EUR A	Jan 1999 - Dec 2008	202	1568024	12.9
**Gyurus et al**	Hungary, Nationwide	EUR C	Jan 1989 - Dec 2009	115	1306818	8.8
**Habeb et al**	Saudi Arabia, Al-Madinah	EMR B	Jan 2004 - Dec 2009	115	672060	17.1
**Harjutsalo et al**	Finland, Nationwide	EUR	Jan 1990 - Dec 1994	520	1609907	32.3

		A				
		EUR A	Jan 1995 - Dec 1999	617	1562025	39.5
		EUR A	Jan 2000 - Dec 2005	910	1713748	53.1
**Harjutsalo et al**	Finland, Nationwide	EUR A	Jan 2006 - Dec 2011	489	889091	55
**Jarosz-Chobot et al**	Poland, Upper Silesia	EUR B	Jan 1989 - Dec 1997	67	239093	3.11
**Jarosz-Chobot et al**	Poland, Upper Silesia	EUR B	Jan 2000 - Dec 2005	153	1597077	9.58
**Joner et al**	Norway, Nationwide	EUR A	Jan 1989 - Dec 1998	385	2935370	13.1
**Kadiki et al**	Libya, Benghazi	EMR B	Jan 1991 - Dec 2000	38	14813	2.6
**Koton et al**	Israel, Nationwide	EUR A	Jan 1997 - Dec 2003	227	4540000	5
**Lee et al**	Singapore, Nationwide	WPR A	Jan 1992 - Dec 1994	18	750000	2.4
**Levy-Marchal et al**	France, Aquitane, lorraine , Upper & lower Normadie	EUR A	Jan 1988 - Jan 1990	46	1121951	4.1
**Lipman et al**	USA, Philadephia	AMR A	Jan 2000 - Dec 2004	60	98161	12.2
**Mazzella et al**	Italy, Liguria	EUR A	Jan 1987 - Dec 1991	23	275582	8.37
**Michalkova et al**	Slovakia	EUR B	Jan-Dec 1990	12	412371	2.91
		EUR B	Jan-Dec 1991	23	403509	5.7
		EUR B	Jan-Dec 1992	26	397554	6.54
**Mitchalkova et al**	Slovakia	EUR B	Jan-Dec 1995	35	348606	10.04
		EUR B	Jan-Dec 1999	57	497817	11.45
		EUR B	Jan-Dec 2000	47	467662	10.05
**Neu et al**	Germany, Baden-Wuerttemberg	EUR A	Jan 1987 - Dec 1993	248	3594203	6.9
**Panamonta et al**	Thai, Northeastern region	SEAR B	Jan 1996 - Dec 2005	40	7017544	0.57
**Pinelli et al**	Italy, Vinetto	EUR A	Jan 1993 - Dec 1994	30	193125	7.2
**Pishdad**	Iran	EMR B	March 1991 - March 1996	53	463000	2.3
**Pronina et al**	Russia, Moscow	EUR	Jan 1996 - Dec 2005	269	3898551	6.9

		C				
**Pundziute-Lyckå et al**	Sweden, Nationwide	EUR A	Jan 1983 - Dec 1998	1625	8575198	18.95
**Radosevic et al**	Slovenia, Nationwide	EUR A	Jan 1998 - Dec 2010	126	1205522	10.5
	Srpska, Bosnia and Herzegovenia	EUR B		62	1160927	5.3
**Rueda et al**	Mexico, Veracruz-Boca del Rio	AMR B	Jan - Dec 1992	2	46526	0.29
**Roche et al**	Ireland, Nationwide	EUR A	Jan - Dec 2008	61	324468	18.8
		EUR A	Jan - Dec 2009	65	335052	19.4
**Roche et al**	Ireland, Nationwide	EUR A	Jan - Dec 1997	27	250000	10.8
**Schranz**	Malta, Natowide	EUR A	Jan 1990 - Dec 1996	30	270270	11.1
**SEARCH study group***	USA, Nationwide	AMR A	Jan-Dec 2001	255	829589	31
**Serban et al**	Romania, Nationwide	EUR B	Jan 1992 - Dec 1995	66	1447482	4.56
**Skordis et al**	Cyprus, Nationwide	EMR B	Jan 1990-Dec 1999	31	521008	5.95
		EMR B	Jan 2000 - Dec 2009	55	429017	12.82
**Skrivarhaug et al**	Norway, Nationwide	EUR A	Jan 2004 - Dec 2012	299	1557292	19.2
**Smith et al**	USA, Chicago	AMR A	Jan 1994 - Dec 2003	82	1012346	8.1
**Songini et al**	Italy, Sardinia	EUR A	Jan 1989 - Dec 1994	619	1862214	33.24
**Soliman et al**	Oman	EMR B	Jan 1993 - Dec 1994	3	194805	1.54
		EMR B	Jan 1994 - Dec 1995	2	206186	0.97
**Staines et al**	Pakistan, Karachi	EMR D	Jan 1989 - Dec 1993	38	6333333	0.6
**Stipancic et al**	Croatia, Nationwide	EUR A	Jan 1995 - Dec 2003	134	2323316	5.77
**Teeaar et al**	Estonia, Nationwide	EUR C	Jan 1983 - Dec 1998	84	535032	15.7
**Tenconi et al**	Italy, Parvia	EUR A	Jan 1988 - Dec 1992	4	16812	4.7
**Torras et al**	Spain, Madrid	EUR A	Jan 1997 - Dec 2005	53	438017	12.1
**Tuomilehto et al**	Mauritius, Nationwide	AFR	Jan 1986 -Dec 1990	9	468019	1.9

		D				
**Tuomilehto et al**	Finland, Nationwide	EUR A	Jan 1987 - Dec 1992	270	964286	28
**Tuomilehto et al**	Poland, Upper Silesia	EUR B	Jan 1989 - Dec 2005	298	5590994	5.33
**Vehik et al**	USA, Colorado	AMR A	Jan 2002 - Dec 2004	146	993197	14.7
**Verge et al**	Australia, New South Wales	WPR A	Jan - Dec 1990	30	410959	7.3
		WPR A	Jan - Dec 1991	32	410256	7.8
**Wadsworth et al**	UK, Bristish Isles	EUR A	Jan - Dec 1992	387	4161290	9.3
**Wong et al**	China, Hong Kong	WPR B	Jan 1986 - Dec 1990	3	71582	0.8
**Wu et al***	New Zealand, Canterbury	WPR A	Jan - Dec 2003	7	21212	33
**Zung et al**	Israel, Nationwide	EUR A	Jan 2002 - Dec 2007	286	2616500	10.9
